# Intense Resistance Exercise Induces Early and Transient Increases in Ryanodine Receptor 1 Phosphorylation in Human Skeletal Muscle

**DOI:** 10.1371/journal.pone.0049326

**Published:** 2012-11-16

**Authors:** Sebastian Gehlert, Gerd Bungartz, Lena Willkomm, Yüksel Korkmaz, Kurt Pfannkuche, Thorsten Schiffer, Wilhelm Bloch, Frank Suhr

**Affiliations:** 1 Department of Molecular and Cellular Sport Medicine, Institute of Cardiovascular Research and Sport Medicine, German Sport University Cologne, Cologne, Germany; 2 Department of Operative Dentistry, Periodontics and Endodontics, Heinrich-Heine-University, Düsseldorf, Germany; 3 Institute of Neurophysiology, University of Cologne, Cologne, Germany; 4 Outpatient Clinic for Sportstraumatology, German Sport University Cologne, Germany; 5 The German Research Center of Elite Sport, German Sport University Cologne, Germany; Mayo Clinic, United States of America

## Abstract

**Background:**

While ryanodine receptor 1 (RyR1) critically contributes to skeletal muscle contraction abilities by mediating Ca^2+^ion oscillation between sarcoplasmatic and myofibrillar compartments, AMP-activated protein kinase (AMPK) senses contraction-induced energetic stress by phosphorylation at Thr^172^. Phosphorylation of RyR1 at serine^2843^ (pRyR1Ser^2843^) results in leaky RyR1 channels and impaired Ca^2+^homeostasis. Because acute resistance exercise exerts decreased contraction performance in skeletal muscle, preceded by high rates of Ca^2+^-oscillation and energetic stress, intense myofiber contractions may induce increased RyR1 and AMPK phosphorylation. However, no data are available regarding the time-course and magnitude of early RyR1 and AMPK phosphorylation in human myofibers in response to acute resistance exercise.

**Purpose:**

Determine the effects and early time-course of resistance exercise on pRyR1Ser^2843^ and pAMPKThr^172^ in type I and II myofibers.

**Methods:**

7 male subjects (age 23±2 years, height: 185±7 cm, weight: 82±5 kg) performed 3 sets of 8 repetitions of maximum eccentric knee extensions. Muscle biopsies were taken at rest, 15, 30 and 60 min post exercise. pRyR1Ser^2843^ and pAMPKThr^172^ levels were determined by western blot and semi-quantitative immunohistochemistry techniques.

**Results:**

While total RyR1 and total AMPK levels remained unchanged, RyR1 was significantly more abundant in type II than type I myofibers. pRyR1Ser^2843^ increased 15 min and peaked 30 min (p<0.01) post exercise in both myofiber types. Type I fibers showed relatively higher increases in pRyR1Ser^2843^ levels than type II myofibers and remained elevated up to 60 min post resistance exercise (p<0.05). pAMPKThr^172^ also increased 15 to 30 min post exercise (p<0.01) in type I and II myofibers and in whole skeletal muscle.

**Conclusion:**

Resistance exercise induces acutely increased pRyR1Ser^2843^ and concomitantly pAMPKThr^172^ levels for up to 30 min in resistance exercised myofibers. This provides a time-course by which pRyR1Ser^2843^ can mechanistically impact Ca^2+^handling properties and consequently induce reduced myofiber contractility beyond immediate fatiguing mechanisms.

## Introduction

Human skeletal muscle tissue represents the vital organ for human locomotion. As such, it exerts a high plasticity to adapt to various modes of exercise [1]. For example, intense contractions of myofibers (as occurs during exercise) initially result in gradually decreased skeletal muscle contraction properties, including acutely reduced myofiber contractility, which occurs already after few seconds of high intense exercise [2], [3]. More importantly, intense exercise also induces sustained alterations in skeletal muscle contractility that last up to several min post exercise [4], [5]. This phenomenon is described as the slow recovery curve of myocellular fatigue [6] in which a sustained increase in relaxation time between periodic contractions of myofibers can be observed [7]. The molecular mechanisms contributing to these phenomena are still a matter of debate [8]. However, calcium (Ca^2+^) plays a fundamental role in the onset of skeletal muscle fatigue [9], [10], [11], [12] because its oscillation in myofibrillar areas links sarcolemma depolarization to muscle contraction, which is also known as excitation-contraction (EC) coupling [13]. The ryanodine receptor 1 (RyR1) channel complex is mainly involved in facilitating the accurate release of Ca^2+^from sarcoplasmic compartments into myofibrillar areas and triggers the important calcium oscillation that is required for continued myofiber contractions. RyR1 has been observed to be posttranslationally modified at several residues, predominantly by phosphorylation at serine^2843^ (pRyR1Ser^2843^) [13]. This modification contributes critically towards skeletal muscle contraction impairment upon exercise as it destabilizes the closed state of RyR1, thereby increasing the ratio of open channels [13], [14], which, consequently, facilitate uncontrolled Ca^2+^release and inaccurate distribution of Ca^2+^ions in myofiber compartments [14], [15]. Bellinger and co-workers determined that chronic exercise in a mouse model results in increased phosphorylation of RyR1 on Ser^2843^ and, consequently, impaired skeletal muscle contractility [14]. Additionally, total RyR1 levels in human skeletal muscle have been found to decrease in response to bed rest and increase due to long term exercise [16]. However, to date, no data are available concerning the acute phosphorylation level of RyR1 in response to exercise. Increased RyR1 phosphorylation is likely to occur in response to acute and severe resistance exercise as this exercise mode requires a high rate of Ca^2+^-oscillation and induces stress within myofibers. RyR1 phosphorylation might have an impact on Ca^2+^handling structures, transiently increased Ca^2+^levels and also on kinases relevant for mediating its own phosphorylation. Thus, an early increase in RyR1 phosphorylation after exercise could potentially provide a subtle cellular indicator for impaired Ca^2+^handling properties in a sensitive recovery period after exercise without affecting general myofiber contractility. Therefore, we hypothesized that increased phosphorylation of RyR1 at Ser^2843^ will be observed in an early and transient time-course in response to high intense resistance exercise in human skeletal muscle. ATP-dependent ion channels and myosin ATPase enzymes exert high rates of ATP turnover and, thus, energetic stress [17] in contracting myofibers, which consequently induces increased phosphorylation of AMP-activated protein kinase (AMPK). Because this emphasizes a contraction-induced effect, we further postulated that exercise-induced phosphorylation of RyR1 is accompanied by elevated pAMPKThr^172^ levels, indirectly indicating that increased pRyR1Ser^2843^ results from myofiber contractions.

Type I and type II myofibers show distinct mechanic and metabolic properties and offer different recruitment patterns during exercise. A distinct recruitment of type I and II myofibers in response to exercise [18], [19], [20] may induce a different load on these myofibers. This may consequently lead to different magnitudes of stress on downstream effectors which mediate the molecular bases for cellular signaling [21] and EC coupling [13]. Thus, we hypothesized that type I and II myofibers would present with differences in pRyR1Ser^2843^ and pAMPKThr^172^ levels in response to exercise, as would individual fibers within each myofiber population due to unequal recruitment of fibers in exercising skeletal muscle.

A model of maximum eccentric leg extensions on human subjects was used to apply high intense muscular workload. Here, we show that pRyR1Ser^2843^ and pAMPKThr^172^ levels increase significantly in an early and transient manner in both type I and type II myofibers 15 to 30 min post exercise. These data are highly significant as they describe the fiber type-specific response of RyR1 phosphorylation in response to acute exercise bouts and beyond.

## Materials and Methods

### Subjects

7 healthy active, but not exceptionally resistance- or endurance-trained, male volunteers (age: 23±2 years, height: 185±7 cm, and weight: 82±5 kg) participated in the present study. All subjects were informed orally and in writing of the study’s purpose and the possible risks involved before providing written informed consent to participate. The study was approved by the Ethic Committee of the German Sport University Cologne in compliance with the Declaration of Helsinki. Dates and times of exercise and muscle biopsies were documented for each subject. Subjects were assigned an anonymous ID during the study.

### Standardization of Diet and Activity before Testing

Subjects were instructed to refrain from vigorous physical activity two days prior to baseline biopsies as well as prior the main stimulus. After fasting overnight, the subjects were advised to drink one standardized energy drink (Fresubin® protein energy drink, Fresenius Kabi Deutschland GmbH, Bad Homburg, Germany; containing 20 g protein, 24.8 g carbohydrate 13.4 g fat, and providing 1260 kJ) two hours prior the training intervention to carry out training in the fed state. Subjects were allowed to drink water ad libitum.

### Experimental Trials

On the day of the main trial, subjects reported to the laboratory at 07:45 AM. Prior to the resistance exercise bouts, a 5 min warm-up with a workload of 1 W*kg^–1^ on a cycle ergometer was carried out. A combined warm-up and main exercise program was carried out subsequently on an isokinetic leg extension machine. The program was automatically operated by the control unit of the isokinetic device. Range of motion, angle velocity, as well as resting time between exercise sets were equalized for each subject. The first phase featured 3 sets of 4 submaximal (30% of maximum force) concentric and eccentric repetitions and 90 sec resting intervals between the sets. The angular velocity was determined by 65°*sec^–1^ movement speed in concentric and eccentric phases. After a 3 min resting phase, the main stimulus was applied featuring 3 sets of 8 maximal eccentric repetitions with a 3 min rest between the sets. The angular velocity during the eccentric contraction phase was carried out at 25°*sec^–1^. The leg was passively returned to the extended knee position over the concentric phase to the starting position with an angular velocity of 75°*sec^–1^. All subjects were verbally encouraged during the exercise session to perform all 24 contractions with maximum force. The exercise regimen induced approximately 70 sec of maximum eccentric force on skeletal muscle.

After the exercise was completed, subjects walked a short distance (≈ 250 m) to the local outpatient clinic of the German Sport University Cologne. After a local anesthesia was applied to the vastus lateralis at its incision, muscle biopsies were collected from the exercised leg.

### Muscle Biopsies

To prevent potential injury due to damaged muscle tissue under maximum eccentric contractions, baseline biopsies of the vastus lateralis muscle were taken at rest 5 days prior the exercise intervention. On the day of the experimental trial, biopsies were collected 15 min, 30 min and 60 min post exercise. The 3 biopsies were taken 2 cm distal from each other starting 2 cm distal of the resting biopsy. Muscle biopsies were obtained from the middle region of the vastus lateralis between the spina iliaca anterior superior and the lateral part of the patella. Biopsies were gathered 2.5 cm below the fascia using the percutaneous needle biopsy technique [22].

### Tissue Processing

Muscle samples were freed from blood and non-muscle material, embedded in Tissue-Tek (Sakura Finetek, Zoeterwoude, Netherlands), frozen in liquid nitrogen-cooled isopentane and stored at −80°C. For immunohistochemical analyses, 7 µm cross sections of all biopsy time points were mounted in triplicates on Polysine® slides (VWR International GmbH, Darmstadt, Germany), aligned carefully for cross-sectional analysis, air-dried and stored at −80°C until further analysis.

### Immunohistochemical Staining and Analysis of pRYR1 Ser^2843^ and pAMPKThr^172^

Immunohistochemical analysis was conducted as described previously [23]. Cryo-sections were brought to room temperature and subsequently incubated for 10 min at 37°C. Sections were fixed for 5 min in −20°C pre-cooled acetone. Sections were blocked in TBS containing 5% BSA for 1 hour at room temperature. Slides were then incubated overnight at 4°C with monoclonal primary phospho-specific antibodies. Antibodies recognizing phosphorylated RyR1Ser^2843^, (Abcam, Cambrigde, UK) [14], [24], total RyR1 (Abcam, Cambridge, UK), phosphorylated AMPKThr^172^ (Cell Signaling, Beverly MA, USA), total AMPK (Cell Signaling, Beverly, MA, USA) and A4.840 (Developmental Studies Hybridoma Bank, Iowa City, USA), which is raised against adult human slow myosin heavy chain type 1 (MyHC1), were used. Antibodies were diluted 1∶200 (rabbit pRyR1Ser^2843^ and total RyR1), 1∶100 (rabbit pAMPKThr^172^ and total AMPK) or 1∶300 (mouse A4.840) in TBS containing 0.8% BSA. Slides were rinsed 5 times for 5 min each with TBS and then incubated for 1 hour with appropriate polyclonal biotinylated secondary antibodies (DakoCytomation, Glostrup, Denmark) diluted 1∶400 in TBS. Negative controls were processed in the same way as the experimental cross-sections; however, primary antibody incubations were substituted by incubation with 0.8% TBS. Thereafter, slides were incubated for 1 hour with Streptavidin biotinylated Horseradish Peroxidase complex (Amersham Biosciences, Uppsala, Sweden) diluted 1∶400 in TBS. After rinsing, staining was carried out using a 3,3`-diaminobenzidine (DAB) solution (0.09 M phosphate buffer (pH 7.4), 2.2 mM DAB, 7.03 mM ammonium chloride, 0.93 mM nickel sulfate, 10.44 mM ß-D-glucose and 0.000024 mM glucose oxidase). After dehydration, the stained sections were embedded in Entellan (Merck, Darmstadt, Germany) and supplied with a coverslip. Muscle cross sections from all biopsy time points of 1 subject were stained within a single batch using the same antibody dilution and development time to minimize variability in staining efficiency. Duplicate mounted serial sections of each time point were stained on a single slide for MyHC1 with the monoclonal A4.840 and polyclonal pRyR1Ser^2843^ antibodies as well as for pRyR1Ser^2843^ and pAMPKThr^172^. The intensity of myofibrillar staining for pRyR1Ser^2843^ and pAMPKThr^172^ was determined separately for type I and type II myofibers. Additionally, myofiber type-specific levels of total RyR1 and total AMPK were determined separately for each time point. pRyR1Ser^2843^ and pAMPKThr^172^ localized specifically to type I and type II myofibers as did total RyR1 and total AMPK.

Staining of pRyR1Ser^2843^ and pAMPKThr^172^ was additionally carried out in identical myofibers to determine the impact of exercise on both parameters in single myofibers. All slides were examined with a Zeiss Axiophot 200 light microscope coupled to a Sony 3CCD Color Video Camera. The background intensity was equalized for each slide and subject prior to image capturing. Differences in staining intensity between time points were determined as the difference between background intensity and measured sarcoplasmic intensity. Digitally captured images (200x magnification) were analyzed to determine the fiber type. To determine the fiber type-specific phosphorylation pattern in the sarcoplasmic compartment of myofibers, pAMPKThr^172^- and pRyR1Ser^2843^-stained cross sections were compared to the consecutive area, showing the corresponding type I and II myofiber distributions. The specific staining intensity was quantified for each myofiber by selection of the sarcoplasmic region of the myofiber and its subsequent assessment by optical densitometry using the software ImageJ® (National Institutes of Health, USA). Intracellular pRyR1Ser^2843^ and pAMPKThr^172^ content was set as the mean staining intensity. 70±6 myofibers per biopsy time point and subject were analyzed for fiber type-specific pRyR1Ser^2843^ and pAMPKThr^172^ signals.

### Immunofluorescence

Tissue was prepared as described under “Immunohistochemical Analysis”. The following primary antibodies were used: mouse α-actinin (Sigma-Aldrich, Steinheim, Germany; dilution 1∶250) and rabbit pRyR1Ser^2843^ (Abcam, Cambridge, UK; dilution 1∶100). The following secondary fluorescent antibodies were used: goat anti-mouse Alexa488 (Invitrogen, Karlsruhe, Germany; dilution 1∶500) and goat anti-rabbit Alexa555 (Invitrogen, Karlsruhe, Germany; dilution 1∶500). Pictures were taken using a Zeiss confocal laser scanning microscope equipped with a Plan-Neofluar 40x/1.3 Oil DIC objective (LSM 510Meta, Zeiss, Jena, Germany). Alexa488 was exited by an Argon laser and detected using the filter set BP505-530. Alexa555 was exited by a Neon laser and detected using the filter set BP565-615.

### Western Blotting

20 mg of muscle tissue was homogenized in ice-cold lysis buffer (Cell Signaling, Boston, U.S.A.) using a commercially available micro-dismembrator (Braun, Melsungen, Germany) and stored at –80°C for further analysis. The protein content of each homogenate was determined by the Lowry test kit (BioRad, Munich, Germany). 30 µg of homogenate protein was thawed on ice, suspended in buffer (0.5 M Tris-HCl, 10% glycerol, 2% sodium dodecyl sulphate, 5% 2-mercaptoethanol, and 0.05% bromophenolblue), and heated at 95°C for 5 min. Tissue proteins were separated on a 6% acrylamide self-casted minigel using a gel casting system (PerfectBlue Vertical Double Gel Systems, Twin S, PeqLab, Erlangen, Germany) in Tris-glycine electrophoresis buffer (0.25 M Tris-HCl, 1.92 M glycine, 1% [w/v] SDS). After electrophoretic separation, proteins were transferred to a polyvinylidene difluoride (PVDF) membrane (BioRad, Munich, Germany) by semidry blotting (65 min at 12 V, 155 mA, and 3 W) using a semidry blotting system (PerfectBlue Semidry Electro Blotter, Sedec S, PeqLab, Erlangen, Germany). The membranes were blocked in 5% low-fat milk for 75 min. Afterwards, membranes were washed with Tris-buffered saline containing Tween (150 mM NaCl, 10 mM Tris-HCl, 0.05% Tween 20, pH 7.5). Membranes were incubated overnight at 4°C with rabbit polyclonal total AMPK and phosphorylated AMPKThr^172^ antibodies (Cell Signaling, Beverly, MA, USA; dilution 1∶1000) total RyR1 and RyR1Ser^2843^ antibodies (Abcam, Cambridge, UK; dilution 1∶500 total RyR1 and 1∶750 pRyR1 Ser^2843^). After membrane stripping using commercially available stripping buffer (ThermoScientific, Rockford, U.S.A.), membranes were re-incubated overnight at 4°C with mouse monoclonal anti myosin heavy chain-1 (MyHC1) antibody (A4.951 dilution 1∶2000, DSHB, University of Iowa, U.S.A.). Total RyR1 western blot analysis was performed on separate gels and membranes. Respective secondary antibodies (goat anti-rabbit and goat anti-mouse, dilutions in TBST for both 1∶2000) were purchased from Thermo Scientific (Rockford, U.S.A.) and membranes were incubated for 1 hour at room temperature. Proteins were detected by an enhanced chemo- luminescence assay (ECL Kit, Amersham-Life Science, Buckinghamshire, UK) exposed to X-ray film (Kodak X-OMAT Engineering, Eastman Kodak Co., Rochester, NY). Total RyR1 and MyHC1 served as internal loading controls and pRyR1Ser^2843^ signals were normalized to total RyR1.

### Statistics

All data are expressed as means ± standard error of means (S.E.M.). A multifactorial ANOVA for repeated measures with the factors ‘time’ and ‘fiber type’ was applied to determine the differences in the phosphorylation pattern of sarcoplasmic RyR1 content over time. In the case of a significant difference between factors, a Bonferroni post-hoc test was applied to locate the differences in fiber type dependent analysis. Tukeys post-hoc analysis was conducted for the western blotting analysis. Statistical significance was set at p<0.05. Linear regressions between variables were assigned using Pearson’s product moment correlation index.

## Results

### Immunohistochemistry and Localization of RyR1

**Figure 1:**
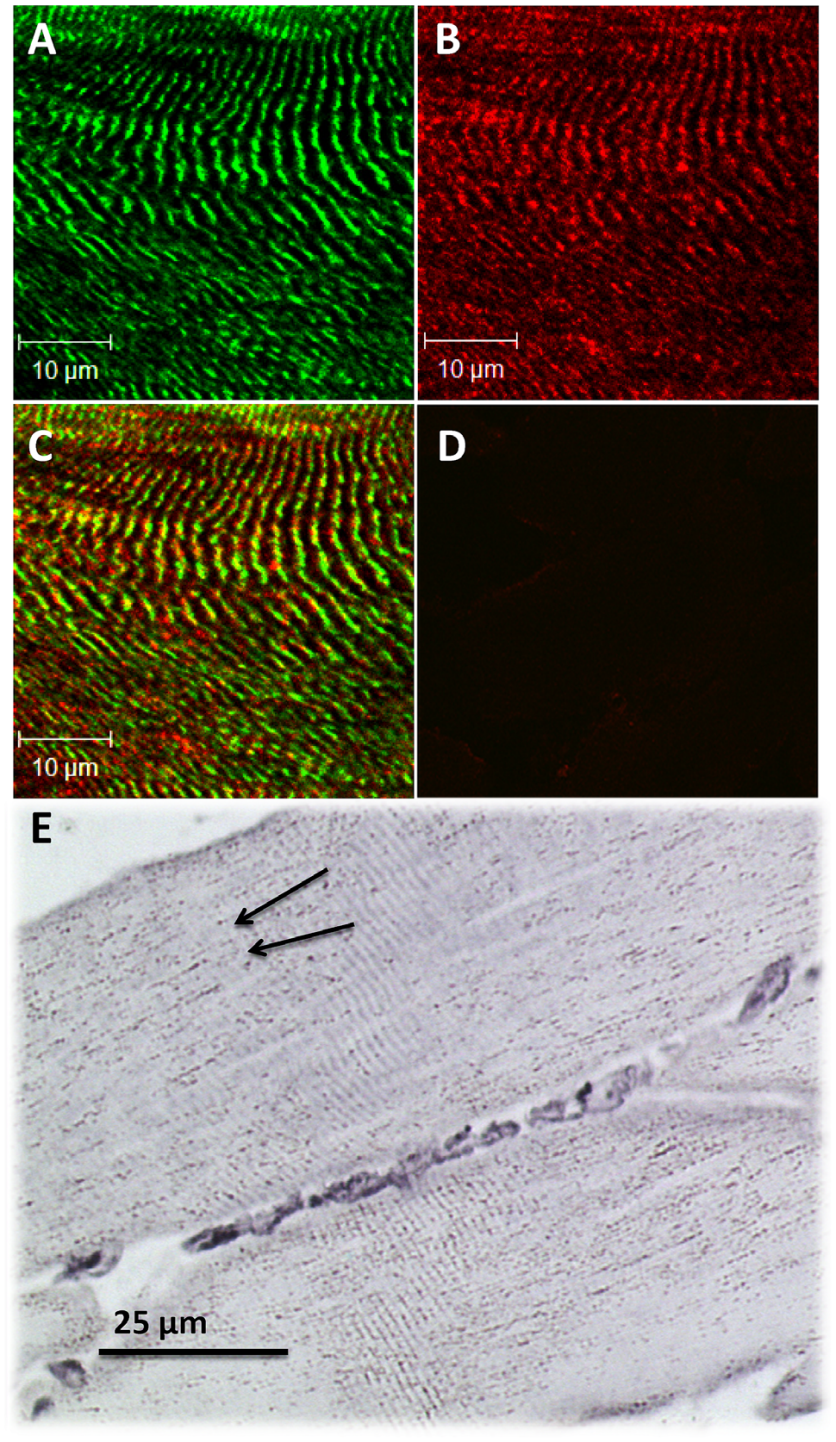
Localization of pRyR1Ser^2843^ in longitudinal and cross-sections of human skeletal muscle myofibers. (**A**) Representative picture of longitudinal arranged myofibers displaying α-actinin staining (Alexa488) and (**B**) pRyR1Ser^2843^ staining (Alexa555) (**C**) Merged picture demonstrates localization of Z-bands (α-actinin, Alexa488) in close vicinity of junctional sarcoplasmic reticuli (pRyR1Ser^2843^, Alexa555). (**D**) Myofiber cross-section displaying an unstained negative control without primary antibody (α-actinin and pRyR1Ser^2843^) incubation. (**E**) Immunohistochemical DAB staining of pRyR1Ser^2843^ in longitudinal skeletal muscle sections (63x magnification). Arrows indicate pRyR1Ser^2843^ localization in cross striated areas.

To demonstrate the pRyR1Ser^2843^ staining specificity, immunofluorescence and DAB staining was performed, which further verified localization of pRyR1Ser^2843^ in junctional sarcoplasmic areas. α-actinin (Figure 1A) and pRyR1Ser^2843^ (Figure 1B) were stained in representative longitudinal cross sections of skeletal muscles. As demonstrated, pRyR1Ser^2843^ was located in cross striated areas in representative longitudinal skeletal muscle sections of immunofluorescence stained (Figure 1C) and DAB stained (Figure 1E) myofibers. Immunofluorescence staining demonstrates Z-bands (α-actinin) located in the proximal vicinity of junctional sarcoplasmic reticuli (pRyR1Ser^2843^) surrounding the t-tubuli which are located in the transitional zone of A- and I-bands. The negative control (Figure 1D) demonstrates that the obtained pRyR1Ser^2843^ signal was specific.

### Determination of Total RyR1 and Total AMPK in Myofibers

**Figure 2:**
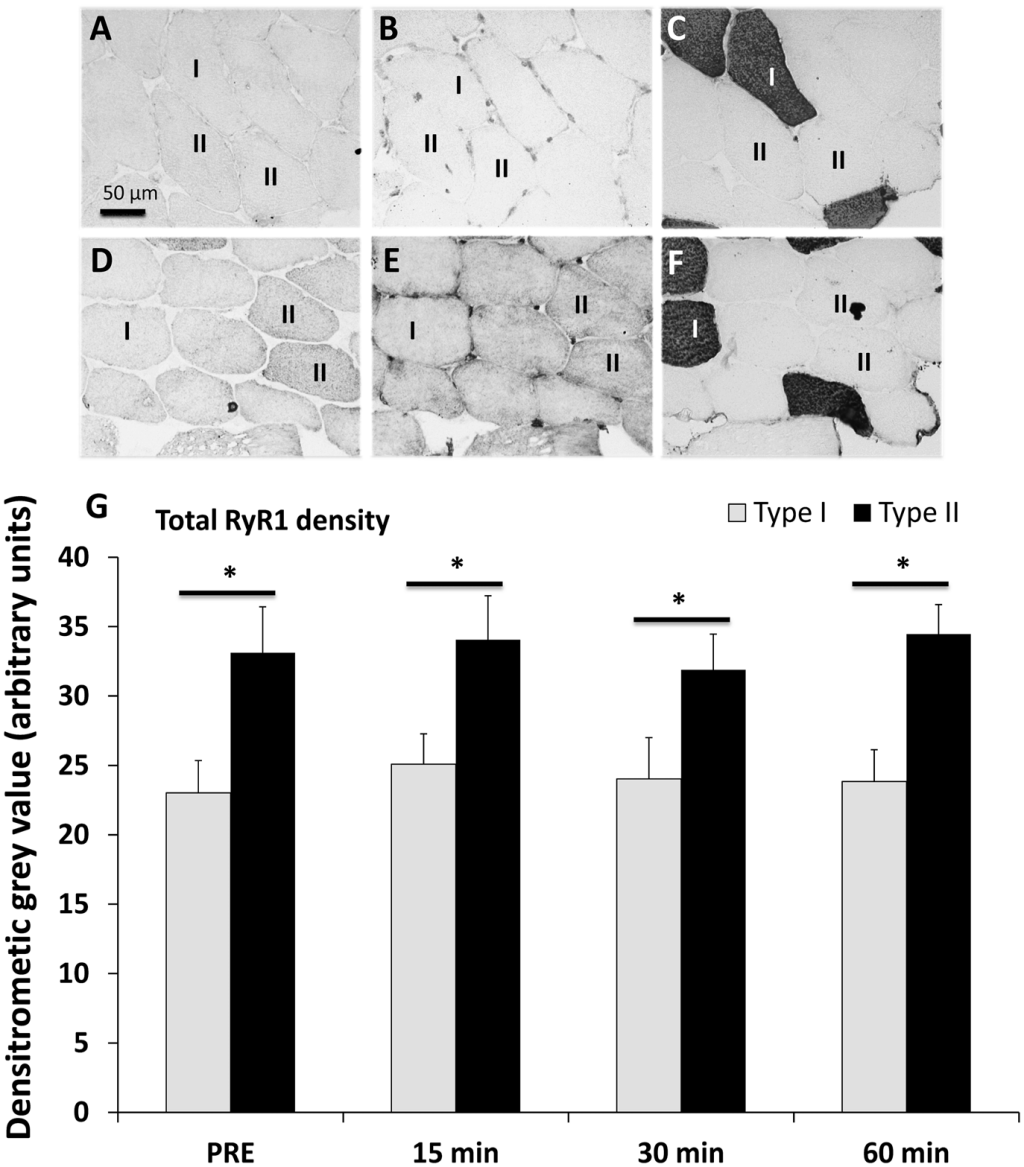
Distribution and localization of total sarcoplasmic RyR1 and total sarcoplasmic AMPK in type I and II skeletal muscle myofibers. Illustration of DAB stained myofiber cross-sections (20x magnification) showing (**A**) total AMPK, (**B**) pAMPKThr^172^ and (**C**) corresponding MyHC1 staining displaying type I myofibers (black) and type II myofibers (unstained). No fiber type-specific differences in total AMPK were observed; Illustration of DAB stained consecutive myofiber cross-sections showing (**D**) total RyR1, (**E**) pRyR1Ser^2843^ and (**F**) corresponding MyHC1 staining displaying type I myofibers (black) and type II myofibers (unstained). As displayed in (**D, F**) total RyR1 density was considerably lower in type I compared to type II myofibers. (**G**) Assessment of total RyR1 density in type I and II myofibers at baseline, 15 min, 30 min and 60 min post resistance exercise. Type II myofibers displayed significantly higher RyR1 densities than type I myofibers (p<0.01). Total RyR1 did not change over the time-course in type I and II myofibers. *headed bars display significant differences between myofibers types (p<0.01).

Salanova and co-workers [16] demonstrated that type I myofibers have less total RyR1 than do type II myofibers in human soleus muscle. To determine whether the human vastus lateralis muscle shows a comparable pattern of total RyR1 abundance and whether acute and severe resistance exercise induces changes in total RyR1 levels over the investigated time-course, we studied the levels of total RyR1 by means of immunohistochemistry. As shown in Figure 2D, the abundance of total RyR1 was significantly different between type I and type II myofibers; however, the amount of total RyR1 in the myofiber types did not change over the time-course (Figure 2G). Importantly, type II myofibers were found to have significantly more total RyR1 than type I myofibers (Figure 2G).

It was recently demonstrated [25] that total AMPK levels do not differ between type I and type II myofibers in human skeletal muscle. Furthermore, the authors did not find any changes in total AMPK levels after exercise [25]. In line with these observations, we did not find any changes in the total amount of AMPK between type I and type II myofibers (Figure 2A), nor did we find any influence of acute resistance exercise on total AMPK levels up to 60 min after exercise (data not shown).

### Resistance Exercise-induced Phosphorylation of pRyR1 Ser^2843^

**Figure 3:**
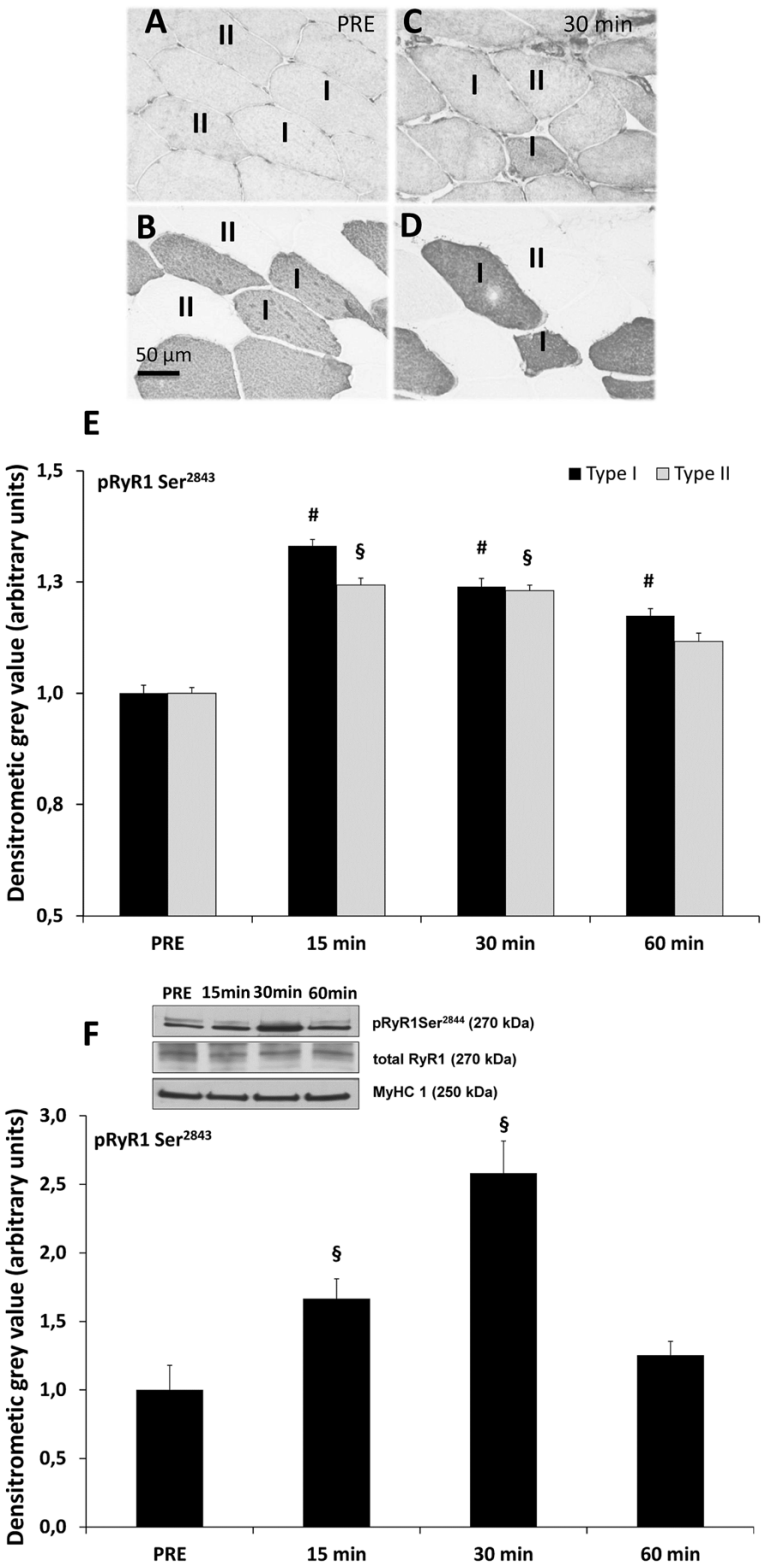
Analysis of sarcoplasmic pRyR1Ser^2843^ in skeletal muscle after resistance exercise. (**A**) pRyR1Ser^2843^-stained myofiber cross-section at baseline (PRE) (20x magnification). (**B**) Consecutive myofiber cross-section specifically stained for type I MyHC1 (A4.840) assigning type I (dark) and type II (unstained) myofibers. (**C**) pRyR1Ser^2843^-stained myofiber cross-section in type I and type II myofibers 30 min post exercise. (**D**) Consecutive myofiber cross-section of figure 3C displaying type I (dark) and type II (unstained) myofibers (**E**) Densitometry analysis of pRyR1Ser^172^ staining in type I and II myofibers cross-sections at baseline as well as 15, 30 and 60 min after resistance exercise. Significantly (p<0.01) increased levels 15 and 30 min post exercise compared to baseline levels in type II **(§)** and up to 60 min in type I **(#)** myofibers. (**F**) Western blotting analysis of pRyR1Ser^2843^ (n = 7). pRyR1Ser^2843^ (band size is observed at 270 kDa according to [14]) was analyzed PRE, 15, 30 and 60 min following exercise in comparison to total RyR1 levels. Myosin heavy chain (MyHC1, 220 kDa) served as an additional internal loading control. (**§**) pRyR1Ser^2843^ significantly different towards baseline levels at 15 min (p<0.05) and 30 min (p<0.01) after resistance exercise.

The differential investigation of pRyR1Ser^2843^ abundance within type I and type II myofibers was carried out by staining consecutive cross-sections for pRyR1Ser^2843^ and type I myosin heavy chain (MyHC1). Figure 3A displays pRyR1Ser^2843^ immunohistochemical staining before exercise and its consecutive MyHC1 stained cross-section showing type I and II myofibers (Figure 3B). Figure 3C shows an increased abundance of pRyR1Ser^2843^ in stained cross-sections at 30 min post exercise. Figure 3D displays its consecutive cross-section stained for MyHC1. Figure 3E shows the phosphorylation levels of pRyR1Ser^2843^ in type I and type II myofibers over the time-course from baseline conditions up to 60 min post exercise in relation to total RyR1 levels. At baseline conditions, no fiber type-specific differences in RyR1 phosphorylation levels were detected. As early as 15 min post exercise, significant increases (p<0.01) in pRyR1Ser^2843^ staining intensity in both fiber types were detected. The RyR1 phosphorylation level in type I and II fibers remained significantly increased (p<0.01) up to 30 min after resistance exercise. pRyR1Ser^2843^ decreased significantly (p<0.01) 60 min after exercise in comparison to the 15 and 30 min time-points, which showed peak phosphorylation in both myofiber types. pRyR1Ser^2843^ phosphorylation levels returned to near-baseline levels in type II myofibers, but remained significantly elevated in type I myofibers (p<0.05) at 60 min post resistance exercise.

To further confirm the findings that pRyR1Ser^2843^ levels are significantly increased at 15 and 30 min post exercise, as determined by quantitative immunohistochemistry, western blots were conducted using whole muscle lysates (Figure 3F). The RyR1 complex has a molecular weight of about 565 kDa [13]. However, it was demonstrated that a band around 270 kDa obtained by western blot analysis is specific for skeletal muscle RyR1 [14], [26]. Total RyR1 (270 kDa) and MyHC1 (220 kDa) served as internal loading controls. Representative examples in Figure 3F show that both total RyR1 and MyHC1 levels remain unchanged within 60 min post resistance exercise. Compared to baseline levels, pRyRSer^2843^ levels increased considerably at 15 min after resistance exercise (p<0.05), peaking at 30 min post resistance exercise (p<0.01) and returning towards baseline levels at 60 min after exercise.

### Resistance Exercise Induced Phosphorylation of pAMPKThr^172^

**Figure 4:**
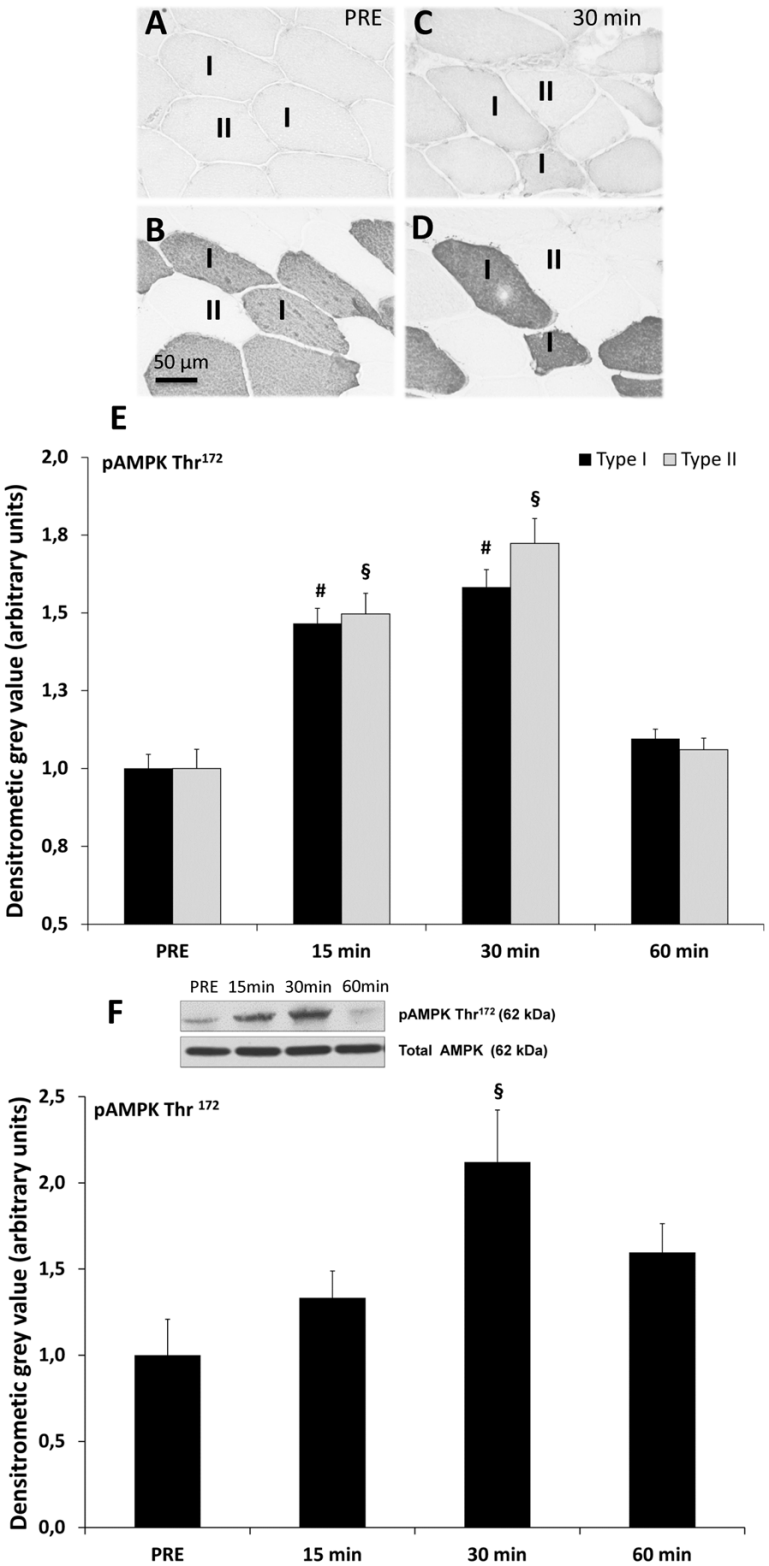
Analysis of sarcoplasmic pAMPKThr^172^ in skeletal muscle after resistance exercise. (**A**) pAMPKThr^172^-stained myofiber cross-section at baseline (PRE) (20x magnification). (**B**) Consecutive myofiber cross-section specifically stained for type I MyHC1 (A4.840) assigning type I (dark) and type II (unstained) myofibers. (**C**) pAMPKThr^172^-stained myofiber cross-section in type I and type II myofibers 30 min post exercise. (**D**) Consecutive myofiber cross-section of figure 3 C. (**E**) Densitometry analysis of pAMPKThr^172^ staining in type I and II myofibers at baseline as well as 15, 30 and 60 min post resistance exercise. Significantly (p<0.01) increased levels 15 to 30 min post exercise compared to baseline levels in type I **(#)** and II **(§)** myofibers. (**F**) Western blotting analysis (n = 7) of pAMPKThr^172^ to the respective time points showing significantly increased pAMPKThr^172^ levels 30 min post exercise (p<0.01) **(§)**.

All subjects completed 3 sets of 8 repetitions of maximum voluntary eccentric knee extensions. pAMPKThr^172^, which has been described to show increased activity upon single bouts of resistance exercise [27], was used as a marker for contraction-induced energetic stress. As shown for pRyR1Ser^2843^, fiber type-specific pAMPKThr^172^ levels were determined in consecutive cross-sections of myofibers stained for pAMPKThr^172^ and MyHC I at baseline (Figure 4A and B) and 30 min post resistance exercise (Figure 4C and D). pAMPKThr^172^ staining intensities are depicted in relation to total AMPK levels. Fiber type-specific phosphorylation of AMPKThr^172^ (Figure 4E) showed significantly increased levels 15 min (p<0.01) post exercise, peaking after 30 min (p<0.01) in both myofiber types. By 60 min post exercise, pAMPKThr^172^ dropped down to baseline levels. Increased pAMPKThr^172^ levels were confirmed by western blotting in muscle homogenates at both 15 and 30 min post exercise (p<0.05, respectively) (Figure 4F) and confirmed to decrease to baseline levels within 60 min post resistance exercise. These data demonstrate the impact of the applied exercise regime on skeletal muscle cellular stress.

### pRyRSer^2843^ and pAMPKThr^172^ Levels in Identical Myofibers

**Figure 5:**
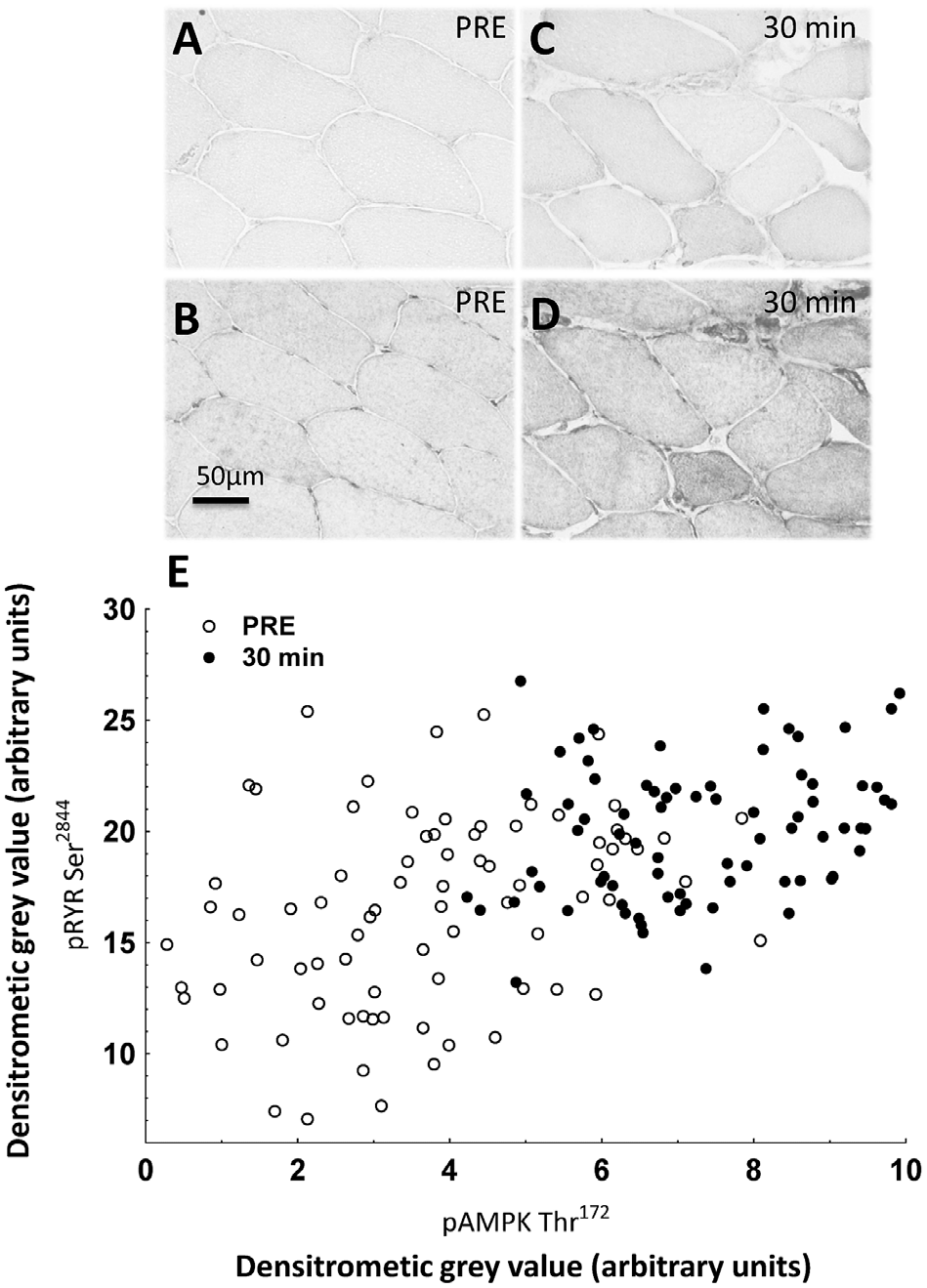
Analysis of sarcoplasmic pRyR1Ser^2843^ and sarcoplasmic pAMPKThr^172^ in identical myofibers at baseline and 30 min post exercise. (**A**) pAMPKThr^172^-stained myofiber cross-section at baseline (PRE) (20x magnification). (**B**) Consecutive myofiber cross-section specifically stained for pRyR1Ser^2843^ at baseline (**C**) pAMPKThr^172^-stained myofiber cross-section 30 min post exercise showing increased staining intensities compared to baseline condition. (**D**) Consecutive myofiber cross-section of figure 4C showing increased staining intensities compared to baseline. (**E**) Distribution of combined phosphorylation levels of pAMPKThr^172^ and pRyR1Ser^2843^ within a population of single myofibers of one representative subject at baseline and 30 min post exercise. Each data point reflects the values of pAMPKThr^172^ and pRyR1Ser^2843^ within a single myofiber to the specified time point. The scatter plot reveals a heterogeneous distribution of combined phosphorylation levels of AMPK and RyR1 within single myofibers at baseline (○). 30 min post exercise the majority of myofibers offered significantly, however gradually increased pAMPKThr^172^ and pRyR1ASer^2843^ phosphorylation levels (•).

We investigated by immunohistochemical techniques the phosphorylation pattern of pAMPKThr^172^ (Figure 5A) and pRyR1Ser^2843^ (Figure 5B) at baseline and 30 min post exercise (Figure 5C and D) in the same myofibers of exercised subjects. The investigation was carried out using multiple consecutive cross sections stained for pAMPKThr^172^ and pRyR1Ser^2843^ from biopsies obtained at baseline and 30 min post exercise. Figure 5E depicts the data points obtained from one representative subject showing the combined staining of pAMPKThr^172^ and pRyR1Ser^2843^ before and 30 min post resistance exercise. As depicted in Figure 5E, control and exercised myofibers presented a broad range of different intensities of both AMPK and RyR1 phosphorylation within myofibers. It is clear, however, that pRyR1Ser^2843^ and pAMPKThr^172^ levels were increased simultaneously in the same myofibers as a consequence of resistance exercise. Hence, the majority of myofibers shifted within 30 min post exercise towards an increased amount of pRyR1Ser^2843^ (Figure 3E) and also pAMPKThr^172^ (Figure 4E) phosphorylation compared to baseline levels. Together, these data demonstrate that RyR1 is phosphorylated in the same fibers as AMPK and, therefore, strongly suggest that RyR1 phosphorylation is a direct consequence of resistance exercise.

## Discussion

Ca^2+^represents an essential ion in skeletal muscle as it primarily mediates the basis of electromechanical coupling in myofibers by its rapid and thorough oscillation between sarcoplasmic and myofibrillar compartments. However, severely contracting skeletal muscle inevitably fatigues in response to an intense workload [4], which leads to gradually reduced contraction properties of myofibers, lasting from seconds up to several minutes after cessation of exercise. This fatigue reduces the ability to perform repeated exercise bouts within a tight time-course [28]. Several cellular factors contribute to this event, including the regeneration of phosphates [29], neuronal factors [30] and Ca^2+^homeostasis [8].

The present study investigated acute effects of eccentric resistance exercise on phosphorylation levels of the skeletal muscle Ca^2+^release channel RyR1. Phosphorylation of RyR1 has been shown to induce “leaky” RyR1 channels [14], impaired myofibrillar Ca^2+^handling and, consequently, reduced contraction performance in chronically loaded mouse skeletal muscle. However, the previous data reveal a lack of knowledge regarding the time-course of RyR1 regulation in response to acute high intense exercise and, in particular, concerning whether different myofiber types are affected to different magnitudes. A primary novel finding of this study is that basal RyR1 phosphorylation levels can be rapidly and transiently increased up to 30 min in human myofibers as a response to 70 sec of maximum eccentric contraction and muscular tension.

We hypothesized that the impact of the applied exercise setup would not affect pAMPKThr^172^ and pRyR1Ser^2843^ levels to an equal extent in the proportion of type I and II myofibers as skeletal muscle constitutes a heterogeneous tissue in which type I and II myofibers are not consistently recruited during exercise [19]. This fact might affect distinct RyR1 and AMPK phosphorylation levels [21] within both populations. pRyR1Ser^2843^ was found to increase significantly in whole skeletal muscle, including both myofiber types. However, relatively higher increases in type I myofibers were observed, which remained elevated up to 60 min post exercise.

To obtain indirect clues for contraction-induced RyR1 phosphorylation, in parallel with the pRyR1Ser^2843^ studies, we investigated phosphorylation levels of AMPKThr^172^, which occurs in response to exercise in skeletal muscle [25]. Its phosphorylation is caused by energetic stress due to increased ATP turnover at ATP-dependent ion channels and myosin ATPase enzymes during electromechanical coupling [17] in myofibers. Here, we determined whether, parallel to elevated pRyR1Ser^2843^ levels, significant increases in pAMPKThr^172^ could be seen in type I and II myofibers up to 30 min post resistance. As we indeed did observe parallel increases in phosphorylation, we conclude that the observed raise in pRyR1Ser^2843^ levels occurred as a response to myofiber contraction. Despite a heterogeneous range of co-regulation of pAMPKThr^172^ and pRyR1Ser^2843^ within the investigated myofiber population (Figure 5E), we surmise that, 30 min after exercise, the parallel increase of both parameters within identical myofibers (Figure 5A–D) reflects a contraction-induced event. We speculate that the observed heterogeneity is due to distinct activity levels of motoneurons at rest and during exercise [19]. Indeed, AMPK and RyR1 phosphorylation can be triggered in response to exercise-induced stress [14], [25]; but it remains unclear whether they directly relate to each other at the molecular level. However, due to the observed paralleled changes in pAMPKThr^172^ and pRyR1Ser^2843^ in the current study, the co-regulation of both molecules, especially those lying in spatial vicinity to each other might be expected. Thus, it will be of future interest to determine whether exercise-induced regulation of AMPK and RyR1 in skeletal muscle myofibers is particularly occurring locally, in mechanically and energetically stressed environment.

Increased AMPK phosphorylation has recently been shown to occur in both myofiber types up to 120 min following endurance exercise [25]. However, the metabolic and mechanical impact of 70 sec of maximum eccentric contractions differs considerably from extended endurance exercise [31], which might have contributed to the shorter time-course of increased pAMPKThr^172^ in the present study. Because AMPK phosphorylation reflects a direct response to contraction-induced energetic turnover [27], this strongly suggests a broad contraction involvement of both type II an especially, type I myofibers in the present study. The observed increase in pRyR1Ser^2843^ takes place in a time-course corresponding to that of AMPKThr^172^ phosphorylation and shows a relatively higher increase in type I compared to type II myofibers up to 60 min post exercise. Our results emphasize the overall higher impact of the applied exercise regimen on type I myofibers. However, besides the observed changes in total RyR1 levels in response to unloading or chronic overload [16], no data are available concerning fiber type-specific responses of RyR1 phosphorylation in response to acute exercise. Our data provide evidence that the observed responses of transiently elevated RyR1 phosphorylation levels within myofibers reflect the impact of strenuous exercise on RyR1 channels within myofibers. Importantly, this can reveal differences in stress-induced regulation of structures involved in Ca^2+^handling in functionally distinct myofiber types. However, molecular differences concerning RyR1 regulation within human myofibers may exist, which to date are not known. This might include a different density of kinases within type I and II myofibers that contribute to RyR1 phosphorylation, e.g CAMKII and PKA, as well as responsible phosphatases that mediate its de-phosphorylation [13]; this would affect the time-course of increased RyR1 phosphorylation in myofibers regardless of any load dependent properties of myofiber types. Thereby, it might be speculated and should be considered in future studies that the observed pRyR1Ser^2843^ elevations are likely be driven by acute activations of CaMKII at Thr^286^ and more importantly by the formation of the catalytic subunit of protein kinase A (PKAc), as both kinases are activated upon acute exercise stimulations [32], [33].

Our data, combined with the described impact of RyR1 phosphorylation on Ca^2+^handling properties by Bellinger and co-workers [14], suggest that RyR1 phosphorylation is increased in response to intense resistance exercise, contributing to a sustained reduction in contraction properties up to 30 min after exercise. This time-course corresponds to a slow recovery period after intense myofiber contractions in which a reduction in myofiber contractility and relaxation ability has been observed [6], [7]. This phenomenon is decidedly one of the most important effects of RyR1 phosphorylation [14], although its phosphorylation has not been linked to the post exercise recovery period after fatiguing muscle contractions.

This is the first study showing the kinetics of increased pRyR1Ser^2843^ after resistance exercise in humans. Our data adds important knowledge regarding the time-course of RyR1 phosphorylation and, importantly, to a time-dependent and partly distinct response in subpopulations of type I and type II myofibers. However, as the difference in RyR1 phosphorylation between fiber types was found to be significant only at the 60 min post exercise time-point, further subpopulation exercise-based studies are needed. Replication of these findings is important as knowing that distinct myofibers may differ in their susceptibility to RyR1 phosphorylation in response to exercise. This what can have considerable impact on the behavior of competing athletes.

The current study does not link the impact of RyR1 phosphorylation within myofibers to decreased myofiber contractility, as multiple biopsies made the investigation of contraction abilities impossible to the investigated time points. However, the current results do extend the known mechanism of RyR1 phosphorylation. Until now, RyR1 phosphorylation has been solely investigated under chronic loading at an early and transient time period after exhausting exercise in human skeletal muscle. Combined approaches, such as EMG derivations, strength testing and multiple biopsies, are needed to investigate the extent to which increased RyR1 phosphorylation alters the contraction abilities of myofibers in general and following repeated training bouts within short recovery periods after exercise. Knowing this time-course would crucially affect a broad spectrum of training behaviors. It will be important to determine whether RyR1 modification can be altered by changes in physical activity between single bouts of exercise. Besides affecting myofiber contractility, pRyR1Ser^2843^-mediated changes in sarcoplasmatic Ca^2+^levels may also offer an important function in exercise-induced Ca^2+^signaling, as elevated sarcoplasmic Ca^2+^levels may trigger enhanced Ca^2+^binding to its targets, including NFAT, Calcineurin and CaMKIV [34]. These factors influence the modulation of myofiber plasticity, e.g. activity-induced myofiber shifting [35] or mitochondrial biogenesis [36], and thus emphasize their importance for exercising skeletal muscle. As each exercise bout induces a transient increase in intracellular Ca^2+^levels, it can be speculated that RyR1Ser^2843^ phosphorylation might also contribute indirectly to early Ca^2+^-induced signaling and, thus, be important for skeletal muscle micro-adaptations.

### Conclusion

The present study indicates that 70 sec of maximum eccentric exercise induces transiently increased phosphorylation levels of pRyR1^2843,^ up to 30 min post exercise, in human type I and II skeletal myofibers. These results provide a sensitized time period in which pRyR1Ser^2843^ can exert a sustained impact on impaired calcium handling properties. This may affect skeletal muscle contraction performance of critically preloaded myofibers in sports beyond immediate fatiguing mechanisms. pRyR1Ser^2843^ may further provide a marker to detect subtle disturbances in skeletal muscle myofiber performance.
